# Research Progress Regarding the Use of Single-Cell Sequencing Technology in Analyzing Tumor Endothelial Cell Pathophysiology

**DOI:** 10.3390/ijms262211128

**Published:** 2025-11-18

**Authors:** Shu Zhao, Siyi Liu, Wenxin Shao, Dong Liu

**Affiliations:** 1 Nantong Laboratory of Development and Diseases, School of Life Sciences, Medical College, Nantong University, Nantong 226001, China; 2 School of Pharmacy, Nantong University, Nantong 226001, China; 19850377216@163.com; 3 Co-Innovation Center of Neuroregeneration, Key Laboratory of Neuroregeneration of Jiangsu and Ministry of Education, Nantong University, Nantong 226001, China

**Keywords:** tumor, single-cell analysis, tumor endothelial cell, heterogeneity

## Abstract

Tumor vascular endothelial cells are essential constituents of the tumor microenvironment, responsible for delivering oxygen and nutrients that are vital for tumor growth and proliferation. As a hallmark of cancer progression, abnormal tumor vasculature contributes to tumor development through multiple mechanisms. Although anti-angiogenic therapies are widely used in the treatment of various cancers, the intrinsic heterogeneity of endothelial cells poses significant challenges regarding therapeutic efficacy. Therefore, further investigation into the heterogeneity of tumor endothelial cells is of paramount importance. The rapid advancement of single-cell sequencing technologies in recent years has facilitated the detailed characterization of heterogeneity among tumor endothelial cells at the single-cell level, thereby fostering a more precise understanding of the functional roles of individual cells within the tumor microenvironment. This technology has become an indispensable tool for investigating the heterogeneity of tumor endothelial cells, offering insights that could inform the refinement of future cancer treatments. In this review, we synthesize findings from the field of single-cell omics to elucidate the heterogeneous characteristics of tumor endothelial cells. We analyze recent advancements in single-cell technology used in the study of tumor cell heterogeneity in terms of both commonalities and distinctive features, covering aspects at the gene and cellular levels. In this review, we provide an overview of recent applications of single-cell sequencing technology in analyzing tumor endothelial cell heterogeneity, offering insights into the development of precise tumor therapies.

## 1. Introduction

**Figure 1:**
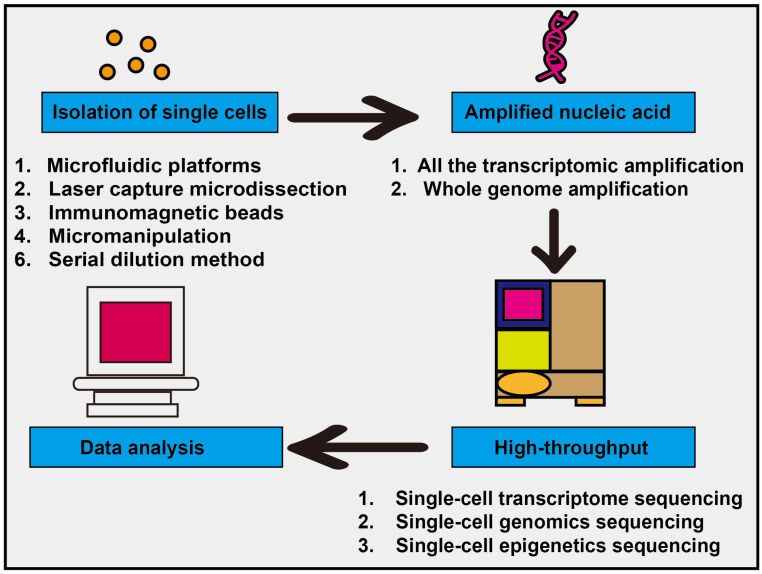
Flowchart of single-cell sequencing technology. The main steps of single-cell sequencing technology include isolating single cells, amplifying nucleic acids, performing high-throughput sequencing, and analyzing data.

**Figure 2:**
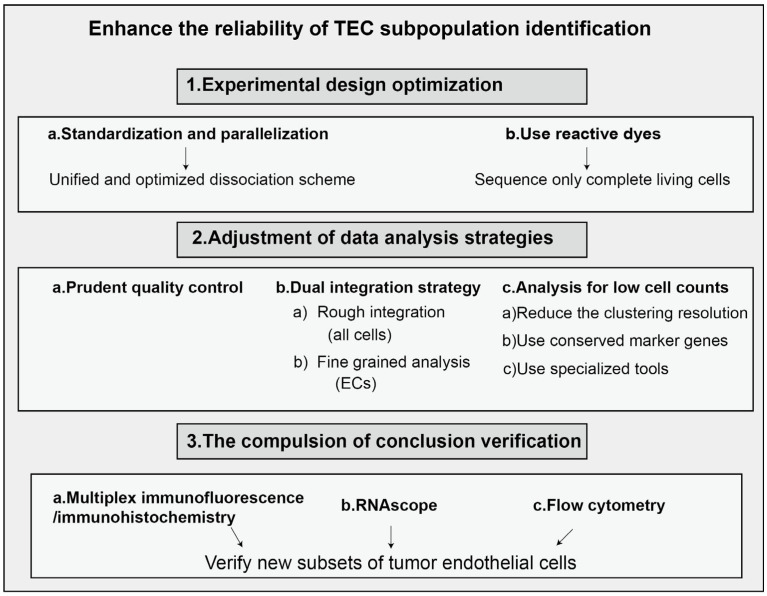
A comprehensive strategy for enhancing the reliability of TEC subpopulation identification in integrated single-cell datasets.

As a type of stromal cell, endothelial cells constitute a critical component of the tumor microenvironment. Endothelial cells are a type of epithelial cell that form a monolayer composed of flat, polygonal cells. These cells interlock with each other, creating a continuous lining that constitutes the inner wall of blood vessels. Common marker genes for endothelial cells include KDR (Kinase Insert Domain Receptor) and CDH5 (Cadherin-5), while universal markers encompass ACKR1 (Atypical Chemokine Receptor 1), PLVAP (Plasmalemma Vesicle-Associated Protein), and IGFBP3 (Insulin-like Growth Factor Binding Protein 3). Endothelial cells play a crucial role in the secretion of vascular regulatory factors, modulation of immune responses, and tissue repair following damage, making them a vital component among the diverse cell types in the human body [1,2]. In contrast to normal endothelial cells, tumor-associated endothelial cells exhibit substantial alterations in morphology and genetics [3,4,5]. These changes encompass the formation of multi-layered endothelial structures, variations in chromosome morphology and number, and gene mutations, among other features [6,7,8]. For instance, in prostate cancer, the overall cell size and nuclear area of tumor-associated endothelial cells are significantly larger than those of normal endothelial cells [9]. These morphological and functional alterations render tumor-associated endothelial cells markedly distinct from their normal counterparts. Moreover, tumor-associated endothelial cells exhibit unique characteristics that differentiate them from normal endothelial cells. Studies have demonstrated that tumor-associated endothelial cells exhibit enhanced resistance to apoptosis and possess transcriptomic profiles that differ substantially from those of normal endothelial cells [10,11]. The tumor vasculature formed by these cells facilitates tumor progression through multiple mechanisms, including the supply of oxygen and nutrients that are essential for tumor growth [12]. Additionally, the tumor vascular system serves as a critical foundation for tumor expansion and proliferation. Research has shown that tumor-associated endothelial cells can employ metalloproteinases to degrade the vascular basement membrane [13]. Thus, in contrast to normal blood vessels, tumor-associated vessels exhibit markedly increased permeability, tortuosity, and disorganization. These structural and functional alterations promote the growth and metastasis of solid tumors, thereby constituting a critical factor contributing to tumor metastasis [14]. Moreover, recent studies have revealed that tumor vasculature not only directly supports tumor growth and metastasis but also plays a pivotal role in tumor immune evasion. Endothelial cells within the tumor microenvironment may compromise the anti-tumor immune response by modulating the infiltration of immune cells [15,16,17]. Tumor endothelial cells comprise several subpopulations that perform distinct biological functions [18]. Heterogeneity is an undeniable characteristic of these cells and presents a significant challenge in their research. Conventional biological experimental techniques are insufficient for conducting precise studies on them. In recent years, the rapid advancement of single-cell sequencing technology has enabled us to analyze the heterogeneity among tumor endothelial cells at a single-cell level. This capability allows for a more precise determination of the functions of individual cell populations within the tumor microenvironment, thereby uncovering novel therapeutic targets and identifying new treatment concepts that facilitate subsequent refined approaches to tumor therapy. The conventional pathway for single-cell sequencing is illustrated in Figure 1. However, the workflow for single-cell RNA sequencing (scRNA-seq) is often discussed in an overly idealized manner, and TEC analysis is one of the areas that is most susceptible to technical artifacts, which introduce several common challenges. Key issues include dropout events, batch effects, and low recovery rates of endothelial cells. In the context of batch effects, for instance, when identifying marker genes that distinguish endothelial cell subsets, apparent markers may instead reflect technical variation associated with batch rather than true biological differences. Furthermore, endothelial cells are typically underrepresented in final datasets due to both the difficulty of tissue dissociation and their relatively low abundance in tumor tissues. Addressing these challenges necessitates an integrated strategy spanning experimental design to data analysis, as illustrated in Figure 2.

This article reviews recent advances in single-cell analysis of endothelial cells, focusing on their common features and heterogeneity across various tumor types from genetic, cellular, and therapeutic perspectives. We summarize and synthesize recent research on the heterogeneity of various tumor endothelial cells utilizing single-cell sequencing technology. This review highlights the advantages of this technology in studying tumor endothelial cells and addresses existing challenges that require resolution. Furthermore, it provides insights and directions for future research on endothelial cells across different types of tumors while anticipating potential solutions to current issues associated with single-cell sequencing technology. This review encompasses both commonalities of and distinctive characteristics inherent to tumor endothelial cells.

## 2. Shared Characteristics: A Comprehensive Single-Cell Analysis of Tumor Endothelial Cells (TECs) Across Various Types of Tumors

The development of single-cell technology has provided unprecedented high-resolution insights into the common characteristics of TECs across various cancer types. These cells not only serve as the structural foundation of the tumor vasculature but also play pivotal roles in regulating the tumor microenvironment (TME). Notable commonalities exist among tumor endothelial cells across different types of tumors. Based on our summary, these commonalities can be primarily observed in the following three aspects: a. at the molecular expression level: alterations in the expression levels of multiple identical genes in tumor endothelial cells from different tumors exhibit consistency; b. at the cellular functional level: for example, a significant proportion of tumor endothelial cells in most tumors contain a subpopulation of tip-like endothelial cells; and c. at the application level: these cells serve as targets for anti-angiogenic therapy (AAT) that is predicated on inhibiting the vascular endothelial growth factor (VEGF) signaling pathway. In terms of commonalities, we summarize and discuss the advancements in single-cell analysis within these three aspects.

### 2.1. At the Genetic Level

In different tumor endothelial cells, genes encoding the stem cell marker CD34, the pro-angiogenic marker CD61, and LOX-1 were found to be upregulated [19,20]. These consistent gene expression changes across various tumor endothelial cells suggest the existence of common characteristics among them. Single-cell analysis of these genes, which exhibit consistent alterations (often upregulation) across various cancer types, offers potential targets for broad-spectrum anti-angiogenic therapy. Conventional approaches require the integration of single-cell datasets from multiple tumor types, involving multiple steps such as data collection and integration, cell type annotation, extraction of endothelial cell subsets, identification of differentially expressed genes between tumor-derived and normal endothelial cells, recognition of commonly altered genes across tumor types, and functional enrichment analysis. For instance, certain cross-cancer-specific markers, such as the tip endothelial cell marker ESM1 [21] and RGCC, are conserved in lung cancer, colon cancer, and glioma and are positively correlated with angiogenic activity. In terms of immune regulation, TECs generally upregulate immunosuppressive molecules (e.g., PD-L1, CD276/B7-H3) and adhesion molecules (e.g., ICAM-1, VCAM-1), thereby forming a “vaso-immunosuppressive axis” through the recruitment of Treg cells or the inhibition of CD8^+^ T cell activity [22,23,24].

Studies have found that certain genes are consistently upregulated in various TECs (Table 1), primarily those involved in angiogenesis, extracellular matrix organization, cell adhesion, and inflammatory responses, as summarized in Table 1. Identifying common gene expression patterns in endothelial cells across different tumor types through single-cell analysis represents an effort to uncover core biological principles from complex datasets. These conserved, upregulated genes across multiple cancer types are not only crucial for understanding the shared mechanisms underlying tumor angiogenesis but also valuable targets for the development of broad-spectrum anti-angiogenic therapies. Such therapies aim to overcome the current clinical challenge of resistance to VEGF inhibitors. For instance, therapeutic agents targeting both VEGFA and ANGPT2 are currently undergoing clinical trials, based on insights into commonly dysregulated pathways [25].

### 2.2. At the Cellular Functional Level

Single-cell transcriptome sequencing (scRNA-seq) technology has provided unprecedented resolution in elucidating the heterogeneity of TECs, leading to the discovery of several tumor-specific endothelial cell subsets that are rare or absent in normal tissues. These specialized subsets are shaped by the tumor microenvironment (TME) and, in turn, support tumor growth, immune escape, and metastasis through the development of dysfunctional vasculature. Major tumor-specific endothelial cell subsets have been identified through single-cell data analysis:

(1) Arterial ECs, venous ECs, capillary-like ECs, and lymphatic ECs: Pan (2024) [26] constructed the most comprehensive pan-cancer vascular single-cell atlas to date, encompassing approximately 200,000 cells across 31 cancer types. The study revealed that venous endothelial cells serve as the initiation site for tumor angiogenesis. Furthermore, three major vascular endothelial cell types were identified: arterial ECs, venous ECs, and capillary ECs. Capillary-like EC numbers are significantly higher (approximately 1.8-fold) in tumor tissue compared to normal tissue, and these cells constitute a major component of the tumor vascular network [26].
(2) APLN^+^ tip cells/TipSI ECs represent the most characteristic tumor-specific subset of endothelial cells and can be considered a “hallmark” of tumor angiogenesis. These cells exhibit high migratory activity, respond to gradients of growth factors such as VEGF, and guide the formation of new blood vessel sprouts. Moreover, they express a variety of cytokines and signaling molecules that facilitate communication with other cell types in the tumor microenvironment, including secreted phosphoprotein 1 (SPP1^+^) macrophages. Usually, tip ECs are expressed during the early phase of blood vessel sprouting, a process that signifies tumor progression and is strongly associated with poor clinical outcomes. The level of its expression may serve as a predictive indicator for the efficacy of anti-angiogenic treatments; an example is bevacizumab, an anti-angiogenic agent that targets VEGF-A. Some recent single-cell studies focusing on tip-like endothelial cells are summarized in Table 2 [27,28,29,30,31].

(3) The endothelial–mesenchymal transition (EndoMT) is a biological process in which endothelial cells acquire mesenchymal cell characteristics, and it has been widely observed in various tumors. Endothelial cells undergoing EndoMT (referred to as EndoMT-like ECs) co-express both endothelial markers (such as PECAM1/CD31 and CDH5/VE-cadherin) and mesenchymal markers (including ACTA2/α-SMA, VIM/Vimentin, FN1/Fibronectin, as well as the transcription factors SNAI1/2 and TWIST1). These cells play a crucial role in promoting tumor cell intravasation into blood vessels [36]. Moreover, they secrete extracellular matrix (ECM) proteins that lead to perivascular matrix sclerosis, thereby impairing drug delivery [37]. This phenomenon is closely associated with poor prognosis in cancer therapy.
(4) There is often a population of endothelial cells exhibiting abnormal function that may contribute to immune regulation. Within the tumor microenvironment, these cells frequently lack key costimulatory molecules, such as CD80 and CD86, which can lead to T cell dysfunction or promote the induction of regulatory T cells (Tregs), thereby facilitating immune tolerance. These endothelial cells highly express MHC class II molecules (e.g., HLA-DRA, HLA-DRB1), as well as costimulatory molecules (e.g., CD74) [38,39].
(5) Immuno-regulatory endothelial cells (ECs) are a subset of endothelial cells that do not directly present antigens but actively modulate immune responses through the expression of various immunosuppressive molecules and adhesion factors. These cells typically exhibit high expression levels of immune checkpoint molecules such as PD-L1 (CD274) [40,41], as well as adhesion molecules including VCAM1, ICAM1, and SELE, which collectively contribute to their immunosuppressive functions. Here, we highlight the pivotal role of immune cells in shaping tumor endothelial heterogeneity. Advances in single-cell technologies have provided deeper insights into the molecular mechanisms underlying immune cell–endothelial cell interactions. Such crosstalk may result in T cells being sequestered at the perivascular regions, limiting their ability to infiltrate the tumor parenchyma effectively. Notably, certain endothelial cell subsets constitutively express high levels of immune checkpoint molecules, including PD-L1, which directly contribute to T cell suppression. Furthermore, cytokines such as IFN-γ, secreted by activated T cells and other immune cells, can induce upregulation of MHC molecules in endothelial cells and enhance their antigen-presenting capacity [34,42]. Collectively, these findings establish immune cells as key regulators driving endothelial heterogeneity within the tumor microenvironment. CXCR4+ tip cells (a predominant angiogenic phenotype) and SELE+ venous endothelial cells (a proinflammatory phenotype) were identified across 19 solid tumor types, revealing significant heterogeneity in endothelial cell composition and functional states among different cancers. Tumor tissues frequently exhibit an increased proportion of CXCR4+ tip cells (promoting angiogenesis) and a reduced presence of SELE+ venous endothelial cells, which may impair immune cell infiltration and subsequently influence treatment response [43]. In-depth investigation of the intricate interactions between immune cells and tumor-associated endothelial cells using single-cell technologies holds promise for identifying novel therapeutic targets and advancing innovative strategies. For instance, research focusing on specific molecules such as CXCR4 is paving the way for new therapeutic approaches.

We have identified cell populations that are characteristic of the majority of TECs. Thus, single-cell data analysis reveals that tumor endothelial cells are not a uniform entity but rather multiple distinct subpopulations with specialized functions. These subsets serve as the primary mediators of abnormal tumor vascular function and the immunosuppressive tumor microenvironment. Identifying these cell populations not only deepens our understanding of tumor biology but also offers valuable tools and strategies for the development of novel vascular-targeted therapies, such as specifically targeting EndoMT or tip cells, as well as for predicting the efficacy of anti-angiogenic and immunotherapeutic interventions.

### 2.3. Application: Anti-Angiogenic Therapy (AAT)

The vascular endothelial growth factor (VEGF) signaling pathway is a major driving force for tumor angiogenesis. Meanwhile, tumor cells frequently facilitate the proliferation and migration of endothelial cells and the formation of blood vessels by generating VEGFA. Additionally, angiogenesis is widely acknowledged as one of the crucial hallmarks of cancer. Hence, AAT, based on blocking the VEGF signaling pathway, has been extensively employed in the treatment of multiple types of cancers [44,45]. Single-cell technology has elucidated the significant heterogeneity of TECs and their intricate interactions with other cellular components within the tumor microenvironment (TME), offering multiple promising targets for the development of novel AAT strategies [46]. Anti-angiogenic therapy inhibits cancer growth by suppressing angiogenesis and the survival of endothelial cells, thereby reducing the density of tumor blood vessels. Given that tumor endothelial cells can reprogram their metabolism and alter their metabolic transcriptome during disease, the metabolism of tumor endothelial cells has emerged as a new target for anti-angiogenic therapy under various pathological conditions.

Nevertheless, due to issues such as insufficient therapeutic efficacy and drug resistance, the success of anti-angiogenic therapy is limited, and there are still numerous aspects that require refinement. The current study highlights the heterogeneity of TECs revealed by single-cell omics research, which has shown that only a small proportion of TECs across most human tumor types exhibit angiogenic activity. This may explain the limited efficacy of AAT. Another study presented findings from the world’s first large-scale single-cell map of tumor vasculature, identifying venous endothelial cells as the origin of tumor blood vessels and demonstrating that APLN+ tip cells can serve as predictors of anticancer drug response [47]. Single-cell technology has enabled the elucidation of the high heterogeneity among TECs and their intricate interactions with other cell types within the tumor microenvironment (TME). These insights offer multiple promising targets for the development of novel AAT strategies. Table 3 summarizes the main novel targets and their functional characteristics for TECs.

Of course, single-cell analysis also reveals some of the deeper reasons underlying the challenges of AAT. 1. Tumor endothelial heterogeneity: TECs exhibit significant heterogeneity both within and across different tumor types. Angiogenic TECs, which are the presumed targets of conventional AAT, constitute only a small fraction in most human tumor types. This limited target availability may be a key factor contributing to the modest therapeutic efficacy and the rapid development of drug resistance. 2. Activation of compensatory pathways: upon inhibition of the primary VEGF/VEGFR signaling pathway, tumors may restore vascular supply through the activation of alternative angiogenic pathways, such as FGF or angiopoietin-2 [48], or by recruiting bone marrow-derived angiogenic cells (BMDCs) [49], ultimately leading to therapeutic resistance. 3. Immunosuppressive tumor microenvironment: as previously discussed, certain TEC subsets possess immunosuppressive properties that actively inhibit anti-tumor immune responses. This characteristic limits the effectiveness of AAT when combined with immunotherapeutic strategies.

Based on single-cell analysis, the development of targeted therapies tailored to specific patient subgroups is essential, particularly through the use of biomarkers to guide treatment decisions. Identifying biomarkers that predict the efficacy of AAT is of critical importance. Single-cell analysis has offered unprecedented insights into the complexity of tumor vasculature. Looking ahead, anti-angiogenic therapy will evolve toward greater precision, combination, and personalization. By targeting specific cellular subsets, inhibiting compensatory signaling pathways, reversing immunosuppressive environments, and utilizing robust biomarkers to identify responsive patient populations, therapeutic resistance can be mitigated, paving the way for more effective cancer treatment strategies [32,50,51,52,53,54,55,56].

## 3. Characteristics of Tumor Endothelial Cells in Different Tumors

Different tumor endothelial cells exhibit a significant degree of intertumoral heterogeneity. This section focuses on the following three critical aspects regarding the characteristics of tumor endothelial cells: the changes in gene expression profiles compared to normal endothelial cells, the origins of tumor endothelial cells across different tumors, and targeted gene delivery therapies utilizing various vectors for endothelial targeting.

### 3.1. Gene Expression Changes in Tumor Endothelial Cells

Single-cell RNA sequencing (scRNA-seq) technology has revealed that TECs are not a uniform entity but rather a highly heterogeneous cell population. This heterogeneity is evident not only within the same tumor but also across different tumor types. Endothelial cells in tumors originating from different organs maintain a degree of organotypic specificity, yet they are also influenced by the tumor microenvironment (TME), acquiring distinct gene expression profiles. For example, RGCC, SPP1 (osteopontin), GPNMB, and APOE are highly expressed in hepatocellular carcinoma (HCC) [57], whereas PLVAP (PV1) [58], CD276 (B7-H3), and ESM1 are predominantly expressed in glioblastoma (GBM). Even within the same tumor, endothelial cells exhibit functional diversity: tip cells, stalk cells, and phalanx cells represent functionally distinct and heterogeneous subpopulations. Moreover, scRNA-seq has uncovered heterogeneity in cellular origin and transcriptional programs, identifying additional endothelial cell subtypes beyond the classical tip/stalk classification that often display distinct immunomodulatory functions. For instance, a subset of immunoregulatory endothelial cells (ECs) highly expresses various immune-related molecules, including PLVAP [59], CD276 (B7-H3), VCAM1, ICAM1 [60], and SELE.

TECs are a functionally diverse and highly heterogeneous population that actively participate in shaping the tumor microenvironment, rather than merely serving as passive victims. Different tumor types, such as liver cancer, brain tumors, and breast cancer, exhibit distinct endothelial cell expression profiles, suggesting that vascular-targeted therapies may need to be cancer-specific. Table 4 describes recently identified markers of endothelial cell subtypes in organ-specific cancers. Emerging therapeutic strategies are expanding beyond traditional anti-angiogenic approaches (e.g., anti-VEGF therapy) to include (a) targeting immunosuppressive endothelial cells, such as through anti-CD276 antibodies [61], to convert “cold” tumors into “hot” tumors; (b) modulating endothelial metabolism, for instance via anti-CD36 antibodies [35]; and (c) targeting specific endothelial cell subsets in distinct organs or functional states to enable more precise and effective interventions.

### 3.2. Distinct Cellular Origins of Tumor Endothelial Cells

TECs originate from multiple sources, and their aberrant heterogeneity is largely attributed to this diverse cellular origin. In some tumors, the tumor endothelial cells originate from stem-cell-like endothelial cells, also referred to as endothelial progenitor cells, which are the physiological origin of endothelial cells. The tumor endothelial cells of these tumors are transformed from normal endothelial cells. In other tumors, the tumor endothelial cells are transdifferentiated from cancer cells or other cells within the tumor microenvironment, a conclusion supported by numerous pieces of evidence. For example, researchers have discovered that, in glioblastoma and lymphoma, the cytogenetic and genomic alterations of tumor endothelial cells are identical to those of cancer cells [66]. Further studies have reported that cancer cells can form pericytes, and other types of cells in the tumor microenvironment, such as dendritic cells and monocytes, also possess the ability to transdifferentiate into endothelial cells [67,68]. Hence, researchers propose that the tumor endothelial cells in these tumors are transdifferentiated from cancer cells or other cells in the tumor microenvironment.

Single-cell analysis techniques, such as scRNA-seq, can be used to infer and verify possible different origins of TECs by decoding their transcriptome signatures. Single-cell analysis tends to make inferences about where the origin is by alignment with known cell types. The core method is unsupervised clustering of scRNA-seq data (such as UMAP, t-SNE), which can identify different endothelial cell subsets. Canonical Marker Expression, Trajectory Inference, RNA Velocity, Clonality Analysis, and other methods have been used to infer EC subset origins. For example, inferred from signature gene expression, true TECs would normally express PECAM1 (CD31), CDH5 (VE-Cadherin), VWF (von Willebrand Factor), CLDN5, and so on [69,70].

Transitional tumor endothelial cells may co-express endothelial cell markers along with markers typically associated with other cell types, such as pericyte markers (e.g., PDGFRB, NG2/CSPG4) and fibroblast markers (e.g., FAP, ACTA2) [71]. This phenomenon reflects a “hybrid” transcriptomic signature. Trajectory inference algorithms, such as Monocle and PAGA, are employed to reconstruct the developmental paths of cellular transitions between distinct states. However, computational findings derived from single-cell analyses must be experimentally validated. Spatial transcriptomics and multiplex immunofluorescence (Multiplex IF) are particularly valuable for confirming the presence of cells co-expressing both endothelial and tumor-associated markers within specific spatial contexts of the tumor microenvironment, thereby validating vascular mimicry, or for identifying cells in transitional states that suggest transdifferentiation [72,73]. For example, in breast cancer, where local proliferation may represent a major aberrant origin, single-cell analysis revealed an endothelial subpopulation of cancer cells that highly expressed progenitor cell markers (CD133, KDR), and RNA velocity analysis indicated their differentiation into mature TECs [74]. In pancreatic cancer, transdifferentiation (e.g., from pericytes or fibroblasts) appears to be the primary underlying abnormal origin [33]. Single-cell analysis identified an endothelial subset co-expressing endothelial markers (CDH5) and mesenchymal markers (PDGFRB, ACTA2), and trajectory analysis revealed a continuous developmental pathway from pericytes to this endothelial subset. A study on laryngeal cancer utilized single-cell transcriptome analysis to further classify endothelial cells within laryngeal tumors into four distinct subpopulations. Among these, vascular endothelial cells characterized by FLT1 expression (vECs-FLT1) were the most abundant. Functional analyses revealed that this subpopulation was strongly associated with angiogenesis regulation, and high expression levels of the THBS1 gene in vECs-FLT1 were correlated with poorer patient prognosis. Whether tumor cells can undergo “transdifferentiation” into vascular endothelial cells has remained a subject of debate in glioblastoma (GBM) research. A study by Yan Zhou’s group at Wuhan University, combining genetic lineage tracing with copy number variation (CNV) analysis of single-cell sequencing data, demonstrated that neither vascular endothelial cells nor pericytes in GBM originate from tumor cells [75].

Single-cell analysis plays a crucial role in elucidating the origin of endothelial cells across different tumor types. On the one hand, this insight facilitates the development of novel combination therapies. For instance, if the vasculature of a particular tumor is predominantly derived from transdifferentiation, therapeutic strategies can be devised to simultaneously target both endothelial cells and their precursor cells, such as pericytes. On the other hand, tumors exhibiting a high prevalence of vascular mimicry or TECs originating from specific cell types are often associated with increased malignancy and poorer clinical outcomes. In this context, single-cell RNA sequencing (scRNA-seq) enables the identification of precise biomarkers for accurate diagnosis and prognosis. Single-cell analysis technology has revealed that tumor endothelial cells are not homogeneous but instead exhibit complex origins, well-defined differentiation trajectories, and highly specialized functional subsets. These insights not only refine and expand our understanding of the fundamental biology of tumor angiogenesis but also offer a valuable framework for overcoming resistance to anti-angiogenic therapies and guiding the development of novel combination treatment strategies.

### 3.3. Endothelial-Targeted Gene Delivery Therapy with Different Vectors

Endothelial-targeted gene delivery therapy is an important approach for the treatment of tumors. Due to the heterogeneity of endothelial cells across different tumor types, the optimal delivery vector varies for each tumor. For example, a novel dendritic macromolecular PAMAM-PEG-SRL nanoparticle system has demonstrated higher transfection efficiency in experiments utilizing C6 glioma cell lines, suggesting improved therapeutic efficacy [76]. Additionally, there are approaches employing poly(β-amino ester) and adeno-associated virus (AAV) for endothelial-targeted gene delivery therapy [77,78]. Different tumor endothelial cells possess distinct optimal delivery vectors tailored to their specific characteristics.

**Figure 3:**
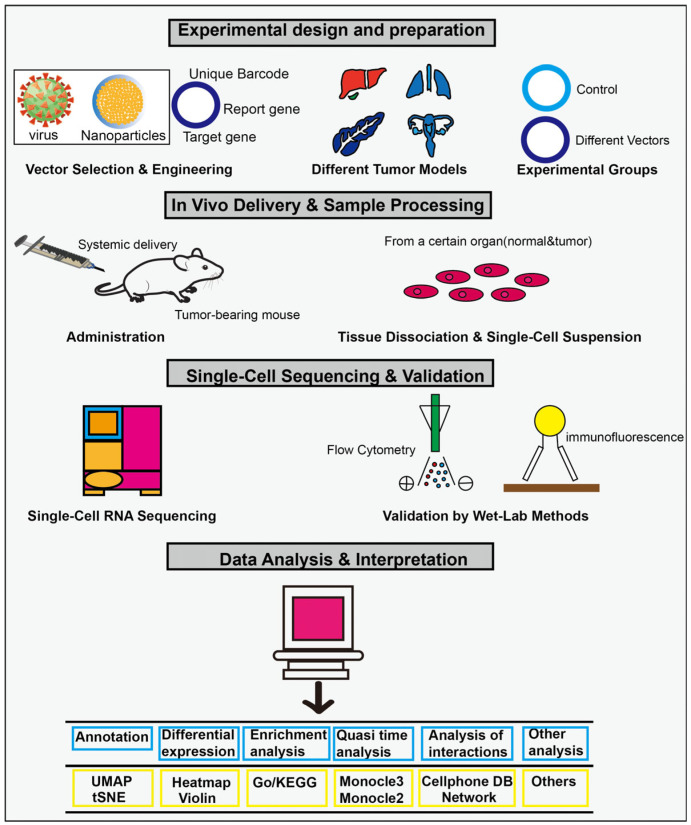
Four sequential stages: experimental design and preparation, in vivo delivery and sample preparation, single-cell analysis and mechanism exploration, and efficacy verification.

Tumor heterogeneity, vector heterogeneity, and endothelial heterogeneity are involved in the process, and single-cell analysis is currently the only technology capable of analyzing the interactions among these three components with such high resolution. In different tumor types, the key challenge lies in leveraging single-cell technology to evaluate and optimize gene delivery vectors so that they can efficiently and specifically target TECs, thereby enabling safe and effective anti-tumor therapy. Single-cell analysis is typically employed to assess the targeting efficiency of various vectors across different tumor types, and this process can be divided into four sequential stages: experimental design and preparation, in vivo delivery and sample preparation, single-cell analysis and mechanism exploration, and efficacy verification (Figure 3). By comparing the performance of different vectors in distinct tumor models, such as glioma and breast cancer, single-cell analysis enables multi-dimensional evaluation, including targeting efficiency, cell specificity, subtype preference, functional impact, and modulation of the immune microenvironment. It is important to note that, in cross-tumor comparisons, the choice of “specificity” in single-cell analysis tools directly affects the reliability of results. The primary objective of cross-tumor endothelial cell comparisons is to minimize non-biological technical variations—such as those arising from sample source and batch effects—while preserving and accurately capturing true biological differences across tumor types, subtypes, or states. For instance, in cell clustering analysis, Seurat offers distinct advantages in integration with clinical data and downstream differential expression analysis, whereas Scanpy demonstrates superior efficiency in handling ultra-large-scale datasets. Quasi-temporal analyses aim to reconstruct dynamic processes such as cell differentiation, which in cancer contexts may reflect tumor stem cell differentiation or T cell exhaustion. Monocle is designed around pseudotime inference based on cell ordering, while Slingshot emphasizes trajectory construction guided by clustering results. To define cell subtype-specific marker genes, a robust differential expression analysis framework should be employed, incorporating multiple testing correction methods such as Bonferroni or FDR. Regardless of the computational tool used, findings from cross-tumor analyses must be validated through independent experimental approaches and confirmed at the protein level using techniques such as immunohistochemistry and flow cytometry.

In the future, obtaining patient samples before and after treatment for single-cell analysis will remain a major challenge in clinical practice. This may necessitate reliance on circulating endothelial cells or more sophisticated biopsy techniques. Integrating spatial transcriptomics with scRNA-seq will be essential to determine the precise localization of transfected endothelial cells within the tumor microenvironment [6,79]. The ultimate goal would be to apply single-cell technologies to characterize tumor endothelial features in individual patients, enabling the selection or design of the most suitable delivery vector to achieve true precision medicine.

## 4. Conclusions and Prospects

Single-cell RNA sequencing (scRNA-seq) technology is a groundbreaking tool that enables analysis of the molecular features of various cells within the tumor microenvironment (TME) at an unprecedented resolution. The heterogeneity of TECs and their complex roles in tumor progression, immune regulation, and treatment resistance are increasingly being uncovered. In this review, we summarize recent advances in scRNA-seq studies of TECs from two perspectives: commonalities and distinctive features. Specifically, TECs share certain common genes, cell subtypes, and therapeutic strategies, yet they also exhibit unique characteristics, including distinct gene expression profiles, specialized cell subtypes, and differential responses to therapies. Several functionally distinct TEC subtypes have been identified through scRNA-seq, each expressing specific marker genes and participating in diverse biological processes. For instance, in bladder cancer, a specialized endothelial cell subtype known as S1 ECs expresses the key marker gene ESM1 [80,81]. In gastric cancer, a unique endothelial cell subtype called Venes-1 is characterized by the expression of IL-33 and MADCAM1 [82,83,84].

The application of single-cell technology to study TEC usually includes the key steps of sample preparation and single-cell isolation, single-cell RNA sequencing, data analysis and cell grouping, in-depth analysis of endothelial cell subsets, and experimental verification. However, simple scRNA-seq may lose the spatial location information of cells in the original tissue. The development of spatial transcriptomics technology perfectly compensates for this deficiency. It allows us to measure gene expression while retaining spatial information on the tissue in situ. Therefore, multi-omics integration and spatial transcriptomics are important for the study of tumor endothelial cell heterogeneity. For example, in osteosarcoma studies, spatial transcriptome technology successfully captured the co-localization of MCAM+ tip-like endothelial cells in the “niche” of tumor cells in metastatic lymph nodes, explaining the metastatic propensity of tumors [30,85]. While multi-omics and spatial transcriptomics technologies advance, there are still several key challenges that significantly impact the study of tumor endothelial cells, potentially confounding the interpretation of their origin and interactions within the tumor microenvironment. For example, data alignment may be inaccurate; while single-cell sequencing enables high-resolution identification of cellular subpopulations (such as the discovery of a specialized subset of tumor endothelial cells), it lacks spatial context. In contrast, spatial transcriptomics preserve positional information but often suffer from low resolution, where a single spot can encompass multiple cells, or from limited throughput. For instance, a “pro-angiogenic endothelial cell” signature identified through scRNA-seq may appear to localize to the tumor core in spatial data. However, if data alignment is imprecise, the signal could originate from normal tissue or blood vessels in the peritumoral region at the tumor margin, potentially creating a false impression of active angiogenesis within the tumor core. Second, noise-related issues can hinder the detection of rare transcripts, particularly when key markers of endothelial cells are low-abundance molecules, thereby compromising the accuracy of cellular origin determination. Moreover, multi-omics combined with spatial data generates extremely large datasets, and the integration analysis algorithms are computationally intensive, demanding substantial computing resources and high algorithmic efficiency. For a highly heterogeneous cell type such as tumor endothelial cells, which exhibit close interactions with the surrounding microenvironment, technical and data-related noise and biases can significantly confound the interpretation of their origins and interaction networks. Acknowledging these limitations and implementing a multi-level, multi-technology validation strategy are essential for deriving robust and reliable scientific conclusions.

Currently, high-throughput single-cell capture is enabled by barcode-based technologies on next-generation sequencing platforms, allowing for the analysis of cellular heterogeneity, such as gene expression variations among distinct subsets of tumor endothelial cells and dynamic changes in cell states during development. In parallel, third-generation sequencing technologies, including PacBio and Oxford Nanopore, offer significantly longer read lengths (up to 10 kb or more), enabling more comprehensive capture of transcript structures. These capabilities make them particularly suitable for investigating complex genomic features such as gene fusions and alternative splicing events. The integration of these approaches has expanded single-cell research from mere gene expression profiling to detailed analysis of gene structure. For instance, CELLO-seq, a recently developed method, achieves high-precision mapping of transposable elements (TEs) and enables their expression and regulatory dynamics to be studied at single-cell resolution [86]. LongCell technology overcomes key challenges in alternative splicing analysis and has uncovered a novel phenomenon: intracellular splicing heterogeneity in highly expressed genes [87]. Furthermore, DeepNanoHi-C leverages deep learning models to address the sparsity and complexity inherent in single-cell 3D genomic data, thereby improving the accuracy of chromatin interaction predictions and the identification of cell-specific structural features [88]. The scMTR-seq technology developed at the University of Cambridge represents a breakthrough by enabling, for the first time, simultaneous profiling of six histone modifications and the whole transcriptome at single-cell resolution. This capability allows researchers to directly correlate epigenetic states with gene expression patterns, offering a powerful approach to uncovering the regulatory mechanisms underlying cell fate decisions [89]. To date, large-scale single-cell transcriptomic studies have independently generated pan-cancer tumor vasculature maps and endothelial cell atlases encompassing dozens of cancer types. These findings have direct clinical implications, as Zhang et al.’s analysis indicates that cancers enriched with CXCR4+ tip cells may exhibit greater sensitivity to anti-angiogenic therapies, whereas those with abundant SELE+ veins may demonstrate enhanced responsiveness to immune checkpoint inhibitors [43]. As these technologies mature and become more widely adopted, DeepNanoHi-C can be applied to investigate how alterations in 3D genome architecture regulate oncogene expression in tumor endothelial cells. Similarly, Longcell or CELLO-seq may be employed to identify critical splicing variants or transposon activation events in endothelial cells—potential candidates for therapeutic intervention. Collectively, these technological advances are elucidating the heterogeneity of tumor vascular endothelial cells with unprecedented precision and paving the way for novel therapeutic strategies.

We have discussed several technical advancements and integrations; however, with respect to the tumor itself, a diverse array of malignant cell clones coexist within the complex tumor microenvironment (TME), which comprises immune cells, stromal cells, and vascular cells. Cell-to-cell interactions, along with the availability of nutrients and oxygen, critically regulate tumor growth, differentiation, invasion, metastasis, and therapeutic response. Single-cell RNA sequencing (scRNA-seq) has uncovered a fundamental dimension of cancer biology by revealing molecular programs that integrate these multifaceted regulatory mechanisms [90]. Extracellular matrix (ECM) remodeling remains a pivotal step in tumor progression, directly influencing endothelial cell infiltration within the tumor microenvironment. Current single-cell sequencing technologies enable the investigation of key biological questions, including the mechanisms underlying ECM remodeling, newly recognized heterogeneity among endothelial cells, ECM–endothelial cell interactions, and their therapeutic implications [91]. Notably, Fan Jia’s team at Fudan University has identified two functionally opposing fibroblast subsets in hepatocellular carcinoma [92]. Meanwhile, Zhang’s research on cross-tissue multicellular coordination offers a more systematic framework, in which the concept of cell modules effectively elucidates the complexity of ECM remodeling [93]. Importantly, ECM remodeling is not merely a structural alteration but an active biological process. For instance, in gallbladder cancer, the CD34+CD90+ endothelial cell subset undergoes endothelial-to-mesenchymal transition (EndoMT) via activation of the TGF-β signaling pathway, acquiring stromal-like properties that enhance its capacity to promote tumor invasion and metastasis [65]. In early-stage gastric cancer, IL-33+ endothelial cell subsets contribute to the progression of advanced disease by upregulating adhesion molecules, enhancing angiogenesis, and facilitating mucosal infiltration. These insights have emerged from the deep integration of single-cell sequencing with multiple cutting-edge technologies, accelerating the translation of findings into clinical applications. For instance, FAP+ fibroblast signatures in renal cell carcinoma serve as potential biomarkers for predicting patient prognosis and resistance to anti-VEGF therapy [94]. By uncovering intricate interactions between the extracellular matrix (ECM) and various cell types, particularly endothelial cells, single-cell sequencing is fundamentally reshaping our understanding of tumor progression mechanisms and paving the way for novel diagnostic and therapeutic strategies.

In summary, single-cell sequencing primarily provides static snapshots of cellular states. Although promising, the application of single-cell technology in studying TECs is still hindered by several challenges. For instance, the workflow from sample preparation to data analysis requires further standardization to ensure reproducibility and comparability across studies. To better understand the dynamic changes and causal relationships in endothelial cell state transitions, it is essential to integrate complementary approaches such as organoid models, lineage tracing, and long-term functional studies. Single-cell RNA sequencing offers unprecedented insights into cellular heterogeneity and interactions [95]. In recent years, single-cell sequencing has opened a new avenue for investigating the heterogeneity of TECs, revealing that these cells are not merely passive “plumbers” but functionally diverse regulators that actively modulate the tumor immune microenvironment and influence therapeutic responses. With ongoing advances in spatial transcriptomics, multi-omics integration, and computational algorithms, such as those used to identify malignant cell populations, it is anticipated that we will be able to construct a more precise map of the tumor microenvironment. This progress may incorporate single-cell analysis into the longitudinal monitoring of patients in clinical trials [96] and ultimately enable targeted therapies directed at specific endothelial cell subtypes, thereby improving outcomes for cancer patients.

## 5. Literature Search

Databases: A systematic search was conducted across several authoritative databases, including Web of Science Core Collection, Scopus, PubMed, and China National Knowledge Infrastructure (CNKI).
Timeframe: The search covered studies published from 2010 to 2025.
Keywords and Search String: An optimized search string was employed using a combination of subject headings and free-text words, connected by Boolean operators (AND, OR, NOT). For instance, the search string used for Web of Science was (“single cell analysis”) AND (“tumor”) AND (“endothelial cell”).

## 6. Literature Screening and Eligibility Criteria

Inclusion Criteria:

(1) Study type: original research articles, reviews.
(2) Subject: involves TECs, focuses on single-cell analysis.
(3) Topic: single-cell studies of TECs.
(4) Language: publications in English and Chinese.

Exclusion Criteria:

Incomplete information: articles for which the full text could not be retrieved or with insufficient data.

## 7. Data Analysis

We employed a thematic analysis for the final included studies. Key information, including author, publication year, study design, sample characteristics, main findings, and conclusions, was extracted from each article. This information was then inductively analyzed, compared, and synthesized to identify and distill the central themes, research trends, points of consensus, and controversies within the literature.

## Figures and Tables

**Table 1:** Some upregulated genes in various TECs.

Gene Symbol	Gene Name	Main Function
VEGFA	Vascular Endothelial Growth Factor A	Core pro-angiogenic signaling molecules
ANGPT2	Angiopoietin-2	Regulating vascular stability, promoting sprouting and proliferation
SPP1	Secreted Phosphoprotein 1	Cell adhesion, migration, and signaling
SELE	E-selectin	Mediating leukocyte adhesion
ICAM1	Intercellular Adhesion Molecule-1	Mediating cell adhesion
VCAN	Recombinant Versican	Remodeling of the extracellular matrix
POSTN	Periostin	Extracellular matrix proteins
COL4A/2	Type IV Collagen	The main component of the basement membrane

**Table 2:** Some recent single-cell studies focusing on tip-like endothelial cells.

Title	Year	Journal	Reference	Datasets	Cell Counts
Single-cell analysis of multiple cancer types reveals differences in endothelial cells between tumors and normal tissues	2023	Computational and Structural Biotechnology Journal	[32]	GSE155698 GSE159115 GSE167297	220,075 cells
Angiogenesis-on-a-chip coupled with single-cell RNA sequencing reveals spatially differential activations of autophagy along angiogenic sprouts	2024	Nature Communications	[33]	GSE155109 PRJNA931762	4693 cells
Protocol for transcriptomic and epigenomic analyses of tip-like endothelial cells using scRNA-seq and ChIP-seq	2025	STAR Protocols	[20]	GSE220509	7529 cells
Single-cell and spatial transcriptomics reveal the key role of MCAM+ tip-like endothelial cells in osteosarcoma metastasis	2025	NPJ Precision Oncology	[34]	GSE162454 GSE152048 GSE21257 HRA007229	129,315 cells
A novel gene signature for predicting outcome in colorectal cancer patients based on tumor cell–endothelial cell interaction via single-cell sequencing and machine learning	2025	Heliyon	[35]	GSE173839 GSE110224 GSE144735 GSE39582 GSE20916 GSE21510 GSE33113 GSE23878 GSE5206 GSE9348	27,414 cells

**Table 3:** The main novel targets and their functional characteristics for TECs.

Types of Targets	Some Associated Cell Subsets/Molecules	Mechanisms of Function	Treatment Strategies
Specific endothelial cell subsets	APLN^+^ tip ECs	Guided new angiogenesis; high expression of pro-angiogenic factors; associated with poor prognosis	To develop drugs specifically targeting APLN^+^ cells, as a biomarker to screen for patients who may respond to AAT
Targets related to immune regulation	MHC-II^+^ ECs	Immunosuppression	Combined with immune checkpoint inhibitors
Subpopulation of pericytes	BASP1^+^ promotes angiogenesis of pericytes (matPCs)	Driven by ER stress, the secretion of VEGF promotes angiogenesis and is associated with poor prognosis	Targeting BASP1^+^ pericytes or their mediated ER stress pathway
Specific signaling pathways	VEGF/VEGFR	The classical angiogenic pathway	Bevacizumab, lenvatinib, and other multi-target TKIs
Interactions between cells	Endothelial cell-immune cell interaction (PODXL-SELL, ICAM1-SPN)	Forming an immunosuppressive microenvironment	Combined immunotherapy to destroy the “vaso-immunosuppressive alliance”
Specific markers of tumor vessels	Universal TEC markers (ACKR1, PLVAP, IGFBP3)	Prevalent in TECs from most tumor types	A potential target or diagnostic tool

**Table 4:** New subtypes of endothelial cells in organ-specific cancers.

Cancer Types	EC Subtypes	Markers
Hepatic carcinoma (HCC)	CD34+ CLDN5+ TECs [62]	CD34, CLDN5, VEGFR2
	CXCL12+ TECs [62]	CXCL12
	PLVAP+ TECs [62]	PLVAP
Pancreatic carcinoma (PDAC)	Endothelioid cancer-associated fibroblasts (endoCAFs) [63]	FAPα, CD144 (VE-cadherin)
Gastric carcinoma (EGC)	IL-33+ ECs [64]	IL-33, CD34, PECAM1
Gallbladder carcinoma (GBC)	CD34+ CD90+ (SAEndo2) ECs [65]	CD34, CD90 (THY1), ESM1
Pan-cancer type	APLN+ tip cells (TipSI) [26]	APLN
	Venous ECs (VenEC) [26]	ACKR1
	Capillary-like ECs (CapEC) [26]	RGCC

## Data Availability

No new data were created or analyzed in this study. Data sharing is not applicable to this article.
